# Influence of Polymer Substrate Damage on the Time Dependent Cracking of SiN_x_ Barrier Films

**DOI:** 10.1038/s41598-018-22105-2

**Published:** 2018-03-14

**Authors:** Kyungjin Kim, Hao Luo, Ting Zhu, Olivier N. Pierron, Samuel Graham

**Affiliations:** George W. Woodruff School of Mechanical Engineering, Georgia Institute of Technology, Atlanta, Georgia 30332 United States

## Abstract

This work is concerned with the long-term behavior of environmentally-assisted subcritical cracking of PECVD SiN_x_ barrier films on polyethylene terephthalate (PET) and polyimide (PI) substrates. While environmentally-assisted channel cracking in SiN_x_ has been previously demonstrated, with constant crack growth rates over short periods of time (<1 hour) during which no substrate damage was observed, the present experiments over longer periods reveal a regime where cracking also develops in the polymer substrate. This time-dependent local cracking of the polymer underneath the channel crack is expected based on creep rupture or static fatigue. Our combined *in-situ* microscopy and finite-element modeling results highlight the combined effects of neighboring cracks and substrate cracking on the crack growth rate evolution in the film. In most cases, the subcritical crack growth rates decrease over time by up to two orders of magnitude until steady-state rates are reached. For SiN_x_ on PI, crack growth rates were found to be more stable over time due to the lack of crack growth in the substrate as compared to SiN_x_ on PET. These results provide a guideline to effectively improving the long-term reliability of flexible barriers by a substrate possessing high strength which limits substrate damage.

## Introduction

The development of ultrabarriers has been motivated over the past decade by their need for organic electronics and thin film photovoltaic technologies. Currently, the technology for ultrabarrier films has reached an effective water vapor transmission rate less than 10^−4^ g·m^−2^ day^−1 ^^1–7^. With recent demonstrations of curved and now foldable organic displays, solar cells and light emitting diodes with radii of curvature down to 30 μm^8^, the understanding of their reliability under mechanical deformation has become paramount to their adoption as a viable technology^9–16^. This is critically important for ultrabarrier films that are made with brittle inorganic thin films and have limited tolerance to strain. Channel cracking is a primary failure mode of concern for ultrabarrier films which has been studied for several barrier film architectures^17–21^. These studies mainly reported crack onset strain values, i.e. the strain at which visual cracks appear in the barrier during bending or stretching test. However, these values may not be sufficient to describe the reliability of brittle barrier films on polymer substrates under mechanical deformation since it does not capture time dependent deformation that can be induced during flexible deformation. This mode of failure can be important for applications such as bendable or foldable electronics that are held in their flexed state of strain for a period of time.

**Figure 1:**
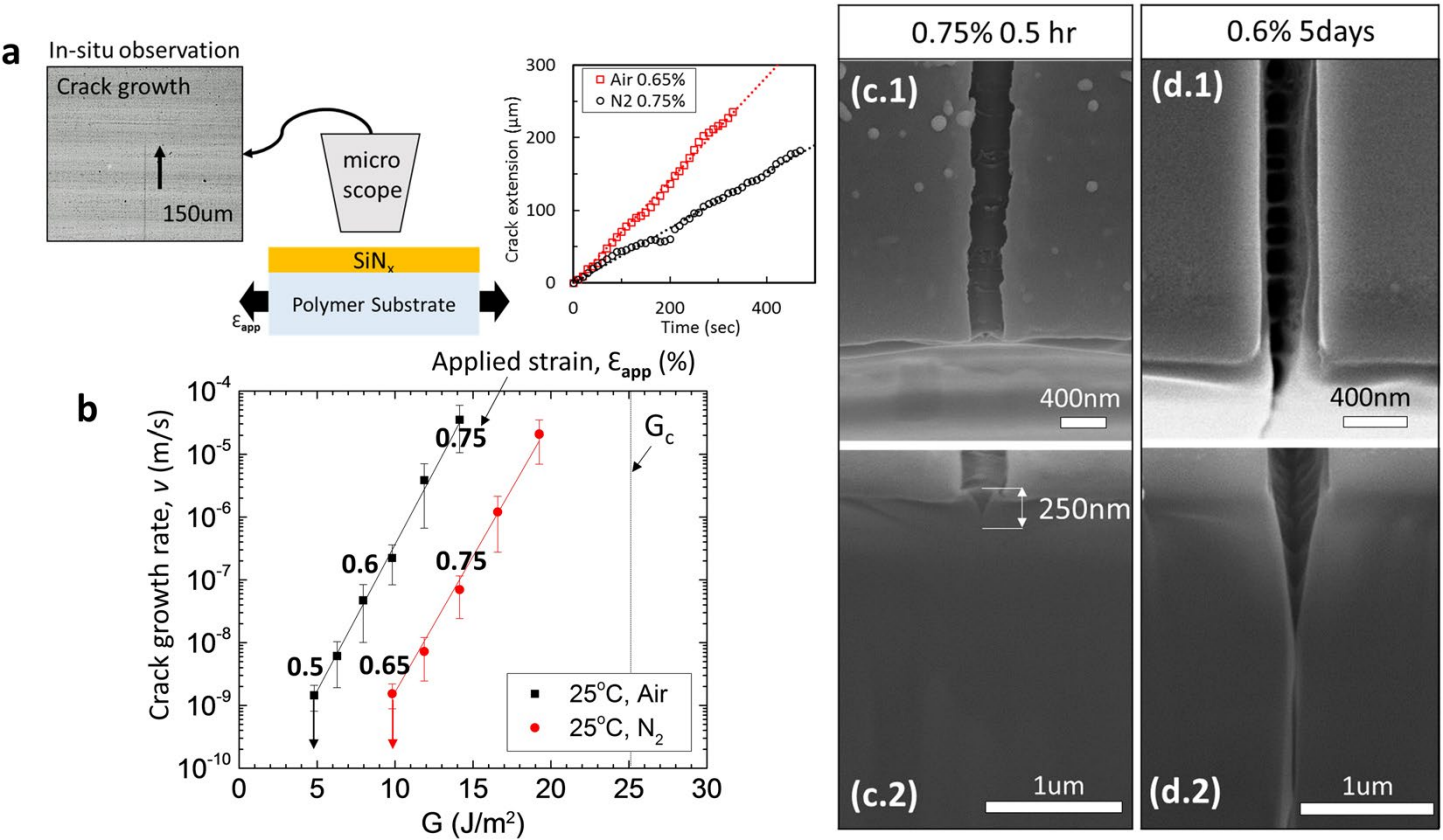
(**a**) Schematic illustration of *in-situ* microscopy of a SiN_x_/PET sample kept at different applied strains, for optical imaging and measurement of channel crack growth and crack extension as a function of time. (**b**) Measured crack growth rate as a function of driving force G for a channel crack in a 250 nm-thick SiN_x_ film in air and nitrogen, respectively. (**c**,**d**) SEM images of SiN_x_/PET cross section (cut by FIB) showing the channel crack in the film as well as the substrate crack beneath the channel crack, under (**c**.1-2) applied strain 0.75% for half an hour and (**d**.1-2) applied strain 0.6% for 5 days.

Recently, we reported the existence of time-dependent subcritical channel crack growth in PECVD SiN_x_ barrier films deposited on polyethylene terephthalate (PET) substrates, as illustrated in Fig. 1a^22,23^. More specifically, we showed that PECVD SiN_x_ films undergo environmentally-assisted cracking and that water molecules are the chemically-active species influencing the crack growth process. As a result, channel crack growth velocity, *v*, is a strong function of environment and driving force for crack extension, *G*, as shown in Fig. 1b. The driving force *G* in Fig. 1b can be calculated accurately using the following equation^24,25^.

1 $${G}_{ss}=Z{E}_{f}^{\ast }{({\varepsilon }_{app}+{\varepsilon }_{res})}^{2}{h}_{f}$$

where *ε*_*app*_ and *ε*_*res*_ are the applied and residual strains in the film; *E*_f_^*^ and *h*_*f*_ are the plane strain elastic modulus and thickness of the film; and *Z* is the dimensionless energy release rate, which depends on the elastic mismatch between film and substrate. The elastic mismatch was characterized by Dundurs’ parameters *α*, *β* given by^26^

2 $$\alpha =\frac{{E}_{f}^{\ast }-{E}_{s}^{\ast }}{{E}_{f}^{\ast }-{E}_{s}^{\ast }}$$

3 $$\beta =\frac{1}{2}\frac{{\mu }_{f}(1-2{v}_{s})-{\mu }_{s}(1-2{v}_{f})}{{\mu }_{f}(1-{v}_{s})+{\mu }_{s}(1-{v}_{f})}$$

where *E*_s_^*^ is the plane-strain elastic modulus of the substrate, *µ*_*f*_ and *µ*_*s*_ are the shear modulus, *v*_*f*_ and *v*_*s*_ are Poisson’s ratio of the film and substrate, respectively. Eq. (1) illustrates the fact that the driving force for channel crack extension is a strong function of the effective substrate constraint (through the coefficient *Z* and therefore *α*. The dependence on *β* is weak.). As such, this equation is only accurate for channel cracking of an isolated crack (whose front width corresponds exactly to the film thickness). For this reason, the data shown in Fig. 1b were only collected for isolated cracks for which no substrate damage was observed (i.e. for periods of time less than 30 minutes).

Previous work has already quantified the effect of substrate cracking on the driving force for channel cracks^27,28^, interaction with neighboring cracks by crack spacing^29,30^ and their application as sensors^31^. However, the influence of substrate cracking on the long-term time-dependent subcritical cracking has not been studied. In this work, we further investigate the time-dependent environmentally assisted cracking in PECVD SiN_x_ barrier films by testing over extended periods of time (i.e. days versus minutes in contrast to our previous study)^22^. Our experimental and numerical results help elucidate the effects of substrate cracking on the driving force (and therefore velocity) of channel cracks in PECVD SiN_x_ barrier layers, which is key to predicting long term damage growth in barrier films under deformation. Specifically, our results highlight various scenarios of increasing, decreasing, or constant crack velocities depending on the substrate cracking configuration and distance to surrounding cracks (i.e. crack density). Details of the experiments and results are described in the following sections.

## Results and Discussion

### Crack Configuration

To test the cracking behavior under extended deformation, PET and PI samples coated with 250 nm of PECVD SiN_x_ were tested under tensile deformation and held at fixed strains. Crack growth rates were measured by *in-situ* optical microscopy. Figure 1c and d show SEM images of focused ion beam (FIB) cross sections of SiN_x_ on PET that were tested at an applied strain of 0.75% for 0.5 h and at 0.6% for 5 days, respectively. The critical onset strain for these films was found to be 0.95% ± 0.2% and subcritical crack growth was observed for these specimens. The SEM images show no PET substrate cracking for the specimen held at 0.75% for 0.5 h (Fig. 1c.1–2). This is consistent with the analysis in our previous study which occurred in a regime without substrate cracking. This is also consistent with constant rates of crack growth in SiN_x_ films measured during short times (~0.5 h) after channel cracking was first observed. In contrast, as clearly shown in (Fig. 1d.1–2), FIB cross section images of the SiN_x_/PET specimen exposed to atmospheric condition for 5 days revealed cracking into the PET substrate directly under the SiN_x_ channel crack. The depth of substrate cracking from the interface with SiN_x_ coating was about 8 µm or 32 times the film thickness. Therefore, the PET substrate undergoes micro-yielding or crazing at the highly stressed channel crack line of the SiN_x_ film, which results in substrate cracking evolving with time via the process known as static fatigue or creep rupture^32^. SEM images of a SiN_x_/PI specimen held for 2 days at 0.75% reveal very little damage in the substrate, while a SiN_x_/PET specimen tested under the same conditions reveal again significant substrate cracking. As will be shown in a later section, the crack growth rates for SiN_x_ on PI are fairly constant, unlike PET for which significant changes in crack velocities are observed over periods of days. It is therefore likely that the substrate damage is responsible for the observed changes in crack velocities, presumably via changing the driving force for crack extension (which influences crack growth rate based on Fig. 1b). Finite element models were used to quantify the effect of substrate cracking on the driving force for crack extension, *G*, in order to explain the observed crack growth rate behaviors over time. For appropriate crack geometries, channel crack in the film, including various depths of substrate cracking in the growing crack/neighboring cracks, was set up in the model. Then, the *G* value at the crack tip of the SiN_x_ layer was calculated through the *J*-integral approach. This driving force was used to predict the growth rate using the *v*-*G* relationship (Fig. 1b).

### Single Crack Growth Rate Behavior

**Figure 2:**
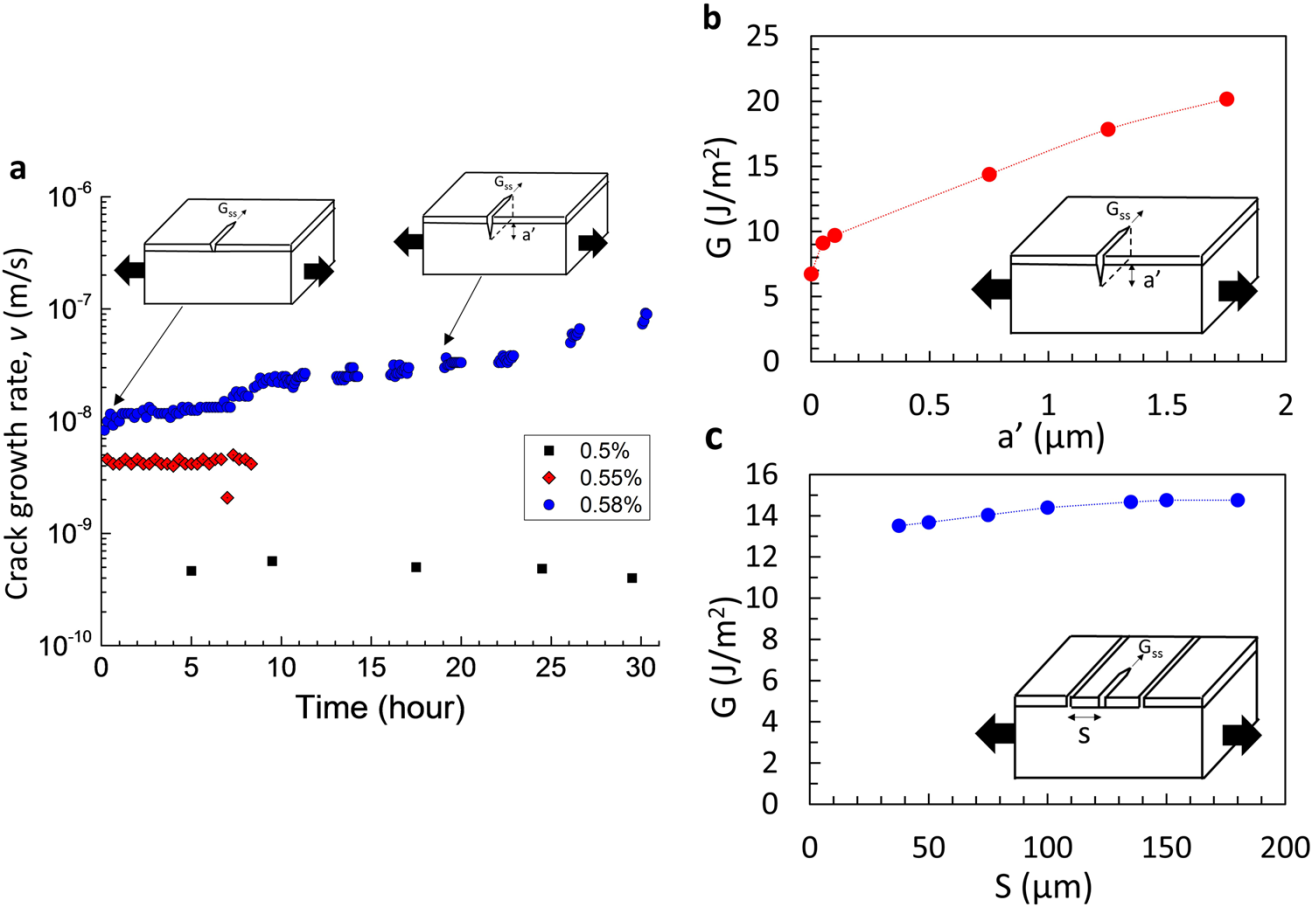
(**a**) Measured crack growth rate in SiN_x_/PET at the applied strains of 0.55% and 0.58% in air. (**b**) Calculated driving force of an isolated crack as a function of substrate cracking depth a’, when the applied strain is 0.58%. (**c**) Calculated driving force of a crack with spacing S to the neighboring long crack on its either side, when the applied strain is 0.75%.

**Figure 3:**
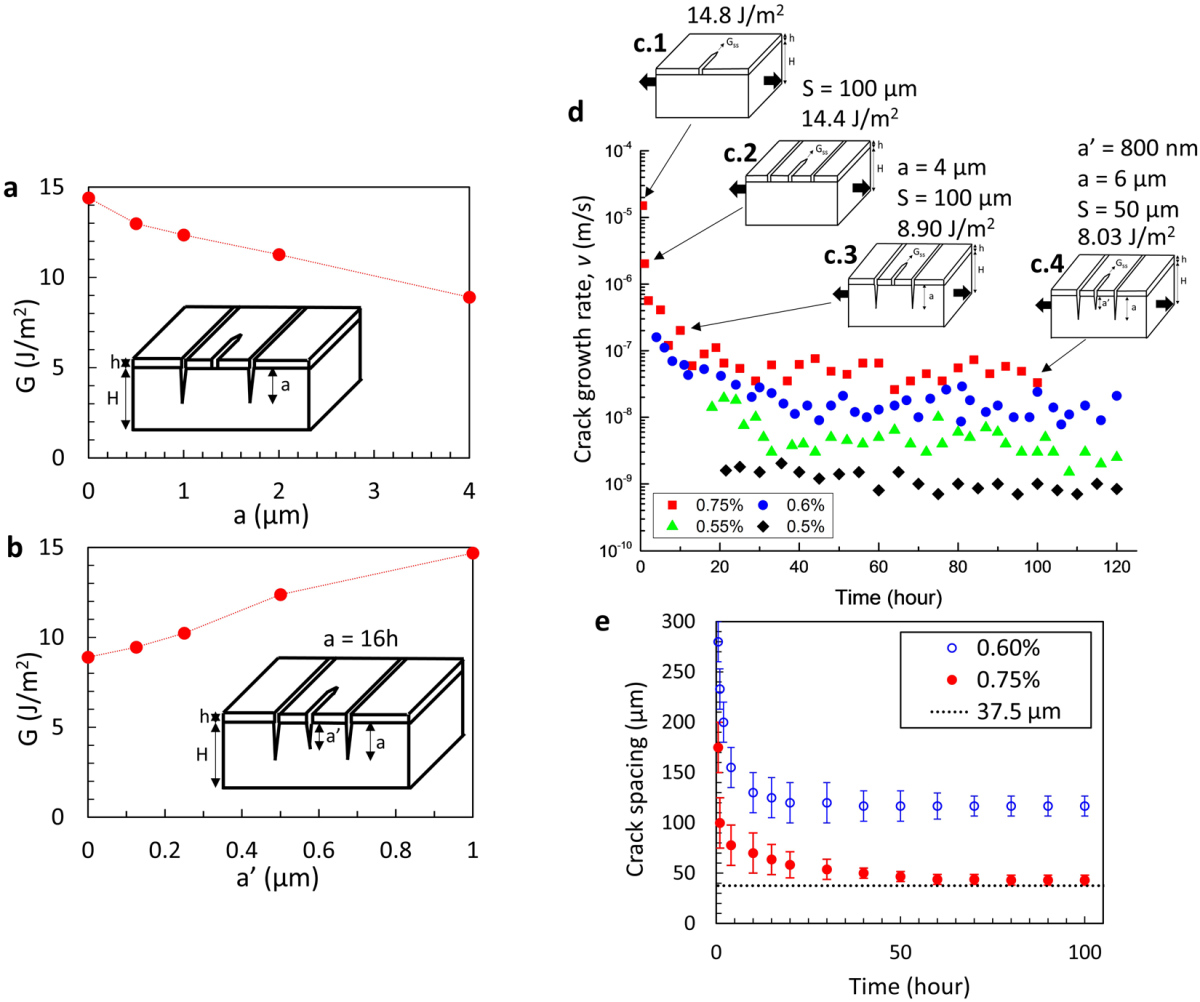
(**a**) Calculated driving force of a crack as a function of substrate cracking depth of neighboring cracks. (**b**) Calculated driving force of a crack as a function of substrate cracking depth a’ in the presence of neighboring cracks with a fixed substrate cracking depth a. Both (**a**) and (**b**) were calculated under the applied strain 0.75%, residual strain −0.15%, crack spacing 100 µm. (**c**) Schematics of different cracking modes in the SiN_x_ film and PET substrate, marked with the associated crack driving force, see text for details; crack spacing S, substrate cracking depth in neighboring cracks a and in growing crack a’ are chosen for each case. (**d**) Measured time dependent crack growth rate of SiN_x_/PET in air, while the applied strain was kept at 0.5, 0.55, 0.6, 0.75%, respectively. For the applied strain 0.6%, the last data point at 120 hours corresponds to the sample in Fig. 1d. (**e**) Crack spacing as a function of time at the applied strain of 0.75% and 0.6%, respectively.

First, we studied the change in crack growth rate for isolated or weakly interacting channel cracks with substrate damage. Finite element modeling results show that the change of driving force of crack growth is less than 1% if the crack spacing is greater than 135 μm (Fig. 2c). Further interactions with neighboring cracks on the driving force as a function of crack spacing can be found in the literature^33^. Consequently, we analyzed the behavior of single cracks by ensuring a crack spacing of at least 135 μm (Fig. 2a). This condition could only be obtained at low applied strains since the crack density quickly increased at higher strains and introduced strong crack interaction effects. We applied the external-load-assisted channel crack growth technique^23^ to accomplish this. Specifically, cracks were initiated quickly by pulling the sample to a strain of 0.75%, followed by a quick strain reduction in order to prevent large crack densities from forming and to find isolated non-interacting cracks. Based on modeling results (Fig. 2b), the development of substrate cracking in the presence of an isolated or weakly interacting crack is expected to increase the driving force due to loss of mechanical constraint to crack opening displacement and therefore the crack growth rate increases (based on Fig. 1b). Thus, an accelerating isolated crack should give evidence of cracking in the underlying substrate whereas steady state isolated cracking would be an indication of no substrate cracking. For low applied strains of 0.5% and 0.55%, the growth rates were measured to be constant throughout the long testing periods as shown in Fig. 2a, an indication that no substrate cracking developed. However, at ε_app_ = 0.58%, the growth rate increased from 8.3 to 100 nm/s over a period of 30 hours (see Fig. 2a), which is an indication of substrate damage. The corresponding increase in crack driving force is estimated to be 33.7% (from 7.03 to 9.40 J/m^2^) from its characterized relationship with subcritical crack growth rates (Fig. 1b). Based on Fig. 2b (showing modeling results for ε_applied_ = 0.58%), substrate cracking between 50 to 100 nm would be required to induce that increase in driving force. This amount of substrate damage in 30 hours is reasonable, given that no damage was observed for 0.5% and 0.55% strain and that substrate cracking was observed at 0.6% strain for 5 days (see Fig. 1d.2). The depth of the substrate crack can be more than 50~100 nm if the substrate cracking occurs in the neighboring cracks with a spacing over 135 µm before it influences the driving force and therefore the crack growth rate. In this case, the crack configuration develops as shown in Fig. 3c.4 (see below, next section).

### Multiple Crack Growth Rate Behavior

Figure 3d shows the results of crack growth rate behavior over periods of time greater than 0.5 h for which substrate damage is expected to play a role. For samples held at strains of ε_app_ = 0.75%, 0.6%, 0.55% and 0.5%, the initial crack growth rates were 15010 ± 8560 nm/s, 159 ± 68 nm/s, 14.1 ± 7.5 nm/s and 1.6 ± 0.35 nm/s, respectively. For the larger rates, the cracks grow and quickly reach the edges of the specimen (width: 5 mm), thus the growth rates of different cracks are measured over 100 hours, especially at 0.75% and 0.6%. These rates decreased over the first ~30–40 h until they reached steady-state values of 50.0 ± 13.9 nm/s, 15.5 ± 5.93 nm/s, 3.74 ± 1.86 nm/s and 1.163 ± 0.394 nm/s, respectively. In the case of applied strain of 0.5%, the growth rate is fairly constant. The initial crack growth rates at each applied strain are in the range of the subcritical crack growth rates in *v*-*G* curve (i.e., crack velocity, *v*, versus the strain energy release rate, *G*) of SiN_x_ thin films as shown in our previous studies, measured for the first half an hour (Fig. 1b)^22,23^. Average crack spacing is plotted as a function of time in Fig. 3e, for tests performed at 0.6 and 0.75%. Along with the evidence of substrate cracking for these extended periods of testing (see Fig. 1d), these data strongly suggest that the observed decreases in crack growth rates are related to increased crack interactions (smaller spacings between cracks for cracks that nucleate and grow in the later stages of the experiments, as more cracks have already propagated through the specimens’ width). In addition, substrate damage keeps developing in channel cracks that have already propagated through the specimen’s width, hence varying amount of substrate damage can be present in neighboring cracks.

Finite element analysis was conducted to provide further insight into the effects of substrate cracking on systems of interacting cracks. For this analysis, a strain of 0.75% was applied to the model where a single propagating crack is interacting with two adjacent cracks. The spacing of the cracks in the model was chosen to be either 50 or 100 μm, depending of the observed crack spacings (Fig. 3e). Film and substrate thicknesses were 250 nm and 125 μm, respectively. To further explore the parametric space, additional calculations were done on a number of crack configurations (single versus multiple cracks, with and without substrate cracking) to elucidate their impact on crack driving forces (Fig. 3a and b). Results show that substrate cracking in the two adjacent cracks reduces the driving force in the growing crack, as the depth into the substrate increases. The driving force decreases by 40% with the increase of substrate cracking depth in the neighboring crack up to 4 µm (Fig. 3a). This is due to the loss of in mechanical constraint in the neighboring cracks as the substrate crack grows in the PET. This has the equivalent effect of closing the growing crack and reducing the energy available for channel crack growth. Figure 3b shows the effect of substrate cracking on the growing crack when the neighboring cracks also induce substrate cracking. When substrate cracking under the growing crack was introduced (up to 1 μm in depth), the crack driving force increased up to 65% while substrate cracking depth in the neighboring cracks was kept at 4 µm (Fig. 3b). The increase in driving force with substrate damage under the growing crack is consistent with the results in the previous section as shown in Fig. 2b.

Based on the modeling results (Figs 2c and 3a,b) and the measured evolution of crack spacing with time (Fig. 3e), a possible scenario for the observed evolution of crack propagation rates can be presented as illustrated in Fig. 3c, with four cases for samples held at 0.75%. The crack driving force value *G* was extracted from modeling and the corresponding growth rate was calculated from the *v*-*G* curve in Fig. 1b. Initially single channel cracks in SiN_x_ develop and the modeling result predicts a driving force of 14.8 J/m^2^ (Fig. 3c.1), corresponding to a crack growth rate of ~75 μm/s based on Fig. 1b. The growth rates for the first 30 mins are on the same order of magnitude, i.e. 15 ± 8.6 µm/s (Fig. 3d). It should be noted that the cracks traverse the full width of the specimen in less than a few minutes, for example, 1 min for 75 μm/s and 5 mins for 15 μm/s, therefore the rates measured after ~0.5 h are for multiple interacting cracks. So, the second case investigated in the scenario after an hour is channel crack growth with reduced crack spacing as depicted in Fig. 3c.2. By adding two adjacent interacting channel cracks (crack spacing 100 µm), the driving force was reduced to 14.4 J/m^2^, corresponding to a slight decrease in crack growth rates (~50 μm/s based on Fig. 1b). Up to this part, substrate cracking is not taken into consideration as evident in the SEM image in Fig. 5b (free from the substrate damage in the PET substrate after an hour at strain 0.75%). However, over time, substrate damage develops first in the existing cracks and the new growing cracks have a lower driving force due to the effect of substrate damage in the neighboring cracks as explained in Fig. 3a, corresponding to the third case in Fig. 3c.3. For example, the crack driving force was further reduced to 8.9 J/m^2^ when a 4 μm crack was present in the PET under the neighboring cracks (Fig. 3c.3, crack spacing 100 µm). This corresponds to a crack growth rate of ~119 nm/s (see Fig. 1b) and is commensurate with the measured rate after 10 hours (see Fig. 3d). At longer times, the cracks were observed to grow much more slowly (~50–100 nm/s) and it took more than 20 hours for cracks to traverse the specimen’s width. Hence the growing cracks whose rates are measured for more than 30 h also undergo substrate cracking (Fig. 3c.4). With 800 nm substrate cracking underneath the growing crack and 6 µm neighboring penetration into PET, the driving force is calculated to be 8.03 J/m^2^. This last case of the scenario occurs when a steady-state growth rate is observed as shown in Fig. 3d after ~30–40 h. This is the result of a balance between additional substrate cracking in the growing crack, i.e. increase of driving force (Fig. 3a) and in the adjacent cracks, i.e. decrease of driving force (Fig. 3b).

### Impact of Environmental Conditions on Crack Growth Rate for Interacting Cracks

**Figure 4:**
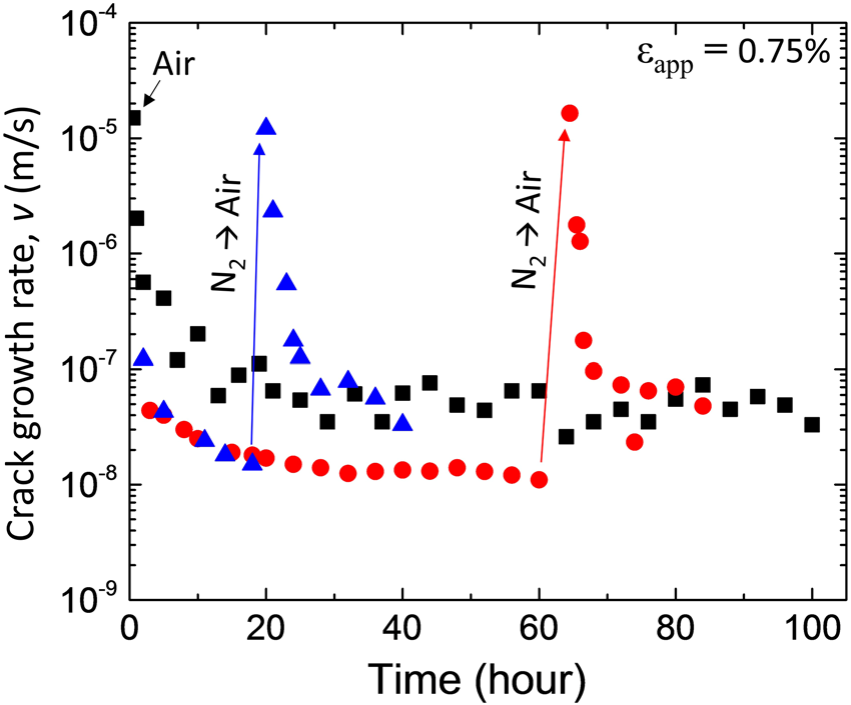
Crack growth rate behavior of SiN_x_/PET at the applied strain of 0.75% subjected to change of environmental condition.

The effect of environment on the long-term crack growth rate behavior was also studied by performing experiments in dry nitrogen for long periods of time before switching to laboratory air. Figure 4 shows that crack growth rate evolution for a specimen tested in air at ε_app_ = 0.75% along with the evolution for two specimens tested at the same applied strain in dry nitrogen before switching to laboratory air after either 20 h or 60 h. In dry nitrogen, the initial crack growth rate is two orders of magnitude lower than in the humid environment, which is consistent with Fig. 1b, highlighting environmentally-assisted cracking. A decrease in rate is also observed in dry nitrogen, with a steady-state value reached after ~20 h, but the magnitude of the decrease is much less than in air. This behavior is attributed to the fact that the density of cracks in N_2_ is two orders of magnitude lower than in air (a few cracks in N_2_ vs. hundreds of cracks in air) and therefore the effect of the interacting cracks (and their associated substrate damage) is much reduced compared to what was described in the previous section. After switching from N_2_ to humid air after 20 and 60 h (for the two specimens shown in Fig. 4), a large increase in the crack growth rate was observed (due to the impact of environmentally-assisted cracking), rising in both cases to rates similar to the initial rates for samples tested in humid air. The newly formed cracks after introducing air behave like a “fresh” specimen tested in air because of the very low density of cracks that formed in nitrogen. The ensuing decrease in crack growth rate is also very similar to that observed in specimens tested in air, suggesting a similar sequence of events described in the previous sections (see Fig. 3c). Lastly, Fig. 4 shows that the steady state rates in nitrogen are only one order of magnitude lower than that in air (while the initial rates were two orders of magnitude lower). The lower relative decrease in nitrogen may be simply due to the lower density of cracks in that environment (leading to lower decreases in driving force), although it is also possible that the humid environment induces more substrate damage that could lead to larger relative decreases in air.

### Crack Growth Rate Behavior on PI Substrates

**Figure 5:**
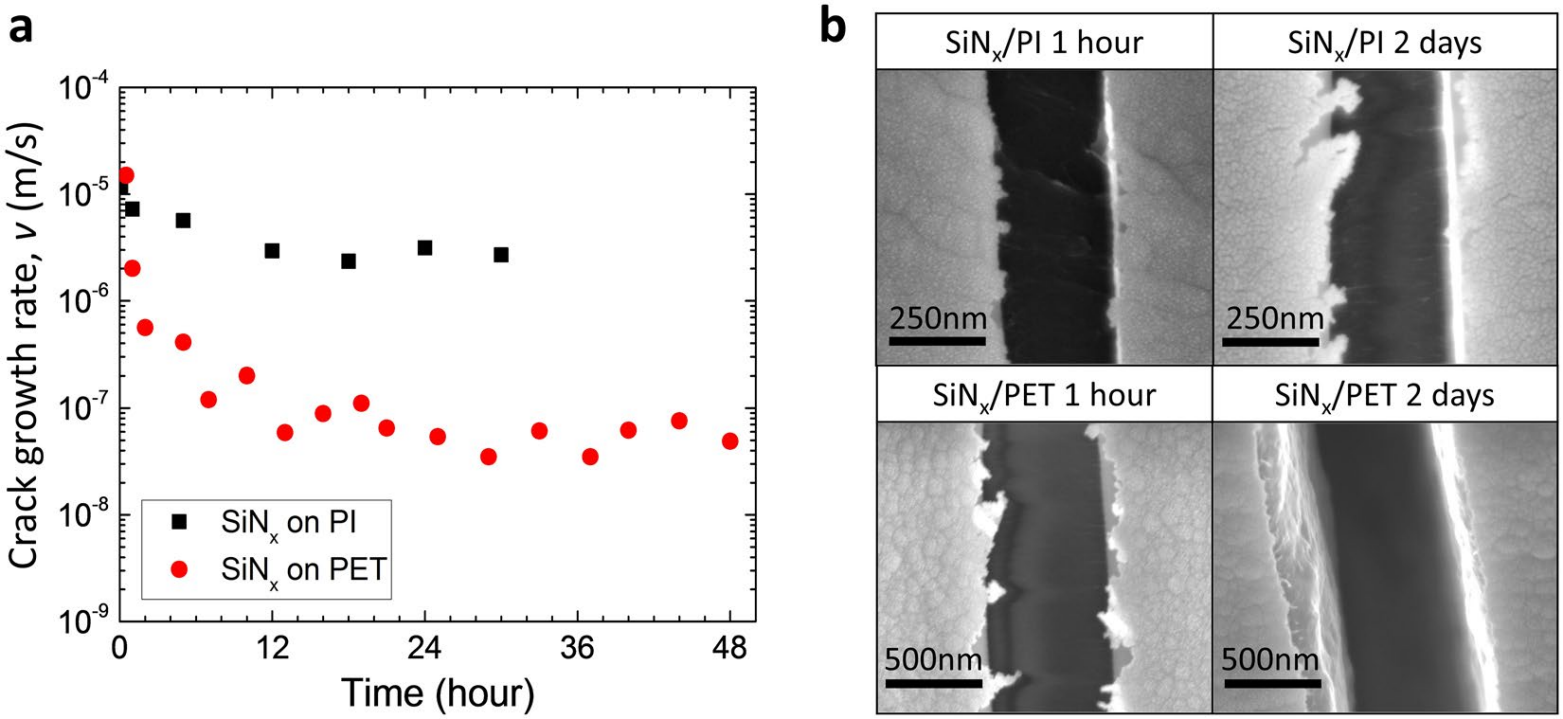
(**a**) Time-dependent crack growth rate behavior of SiN_x_ deposited on PET at the applied strain 0.75% versus on PI at the applied strain 0.85%. (**b**) SEM images of SiN_x_/PET and SiN_x_/PI after 1 hour and 2 days passed, respectively.

Compared to PET, PI has a higher tensile strength (PI: 340 MPa, PET: 170 MPa by ASTM D882) and thus should have greater resistance to substrate damage. Hence, the long-term evolution of crack growth rates in SiN_x_ on PI should be markedly different from that measured with PET, under same initial channel crack driving force, *G*. In order to test a SiN_x_ specimen on PI with the same initial *G* value, the Z parameter from Eq. (2) was evaluated for PI. The moduli of elasticity of SiN_x_ and polymer substrates were determined by nanoindentation and uniaxial tensile testing, respectively^22,23^. The modulus of PI was found to be 7.6 ± 0.17 GPa, higher than PET which has found to be 4.07 ± 0.12 GPa. The elastic mismatch between SiN_x_ and PI was α = 0.880 and between SiN_x_ and PET was α = 0.934 and the corresponding dimensionless energy release rates are Z = 8.62 and Z = 11.8, respectively^34^. Based on these values, a higher applied strain 0.1% was required for PI samples, i.e. 0.85%, to match the crack driving forces between PI (13.8 J/m^2^) and PET (14.4 J/m^2^ for PET at 0.75%). Hence for these two experiments, there is less than 5% difference in the initial driving force. In calculating *G* with Eq. (1), the residual strain is also required. For residual compressive strains (as for PECVD SiN_x_ films), this can be quantified by first straining (in our case up to 0.8%, subcritical value of crack onset strain) a specimen to form a few channel cracks and then catching the applied strain while unloading at which the cracks start closing and become invisible^22^. Residual strains were found to be −0.15% for both PI and PET. As shown in Fig. 5a, the decrease in crack growth rate with time for SiN_x_/PI was much less than that of SiN_x_/PET. In SEM images, substrate cracking was not detected in both of PI and PET after one hour. However, after 2 days, crack penetration was observed in the PET as expected, whereas PI was still free from substrate cracking. A little decrease in crack growth rate of SiN_x_/PI presumably came from inherent damage on the top surface of the substrate. It is important to note that, when both of the substrates are under the same value of applied strain, the number of cracks in SiN_x_/PI was greatly decreased when compared to SiN_x_/PET. For example, at the center of the specimen after 2 days of testing, 255 ± 32 cracks were accumulated in SiN_x_/PET while 21 ± 5 cracks were accumulated in SiN_x_/PI. This certainly demonstrates that depositing barriers on substrates with high tensile strength and therefore resistance to substrate damage is one effective means of improving the reliability of barrier films under long-term mechanical loading.

## Methods

### Sample preparation

250 nm PECVD SiN_x_ (Unaxis PECVD system with radio frequency (rf) parallel plate configuration) films were deposited on commercial heat-stabilized 125 *µ*m thick PET (Dupont Teijin Films Melinex ST505) and on PI (UPILEX-125S) at a temperature of 110 °C, pressure of 1 Torr, 20 W rf plasma and a rate of 10 nm/min. The substrates were laser cut prior to deposition to a size of 5 mm by 50 mm to prevent damage that can occur in trying to cut out samples post deposition.

### Microscopy and image analysis

*In-situ* microscopy from an optical microscope (Edmund Optics, 1312 M 59–365) was used to observe the time-dependent crack propagation on the surface of PECVD SiN_x_ film deposited on laser-cut 50 mm long and 5 mm wide samples using a microtensile testing stage (Linkam Scientific Instruments, TST350). Detailed experimental information is explained in our previous work^22,23^. To observe the substrate cracking, samples were stretched up to a maximum 5 days, i.e. 120 hours, so that a sufficiently large crack could be observed. Tests were performed in a controlled environment as well as laboratory air. Specifically, the environmental condition was switched from dry nitrogen (i.e., 2 ppm) to air (i.e., relative humidity content of 30%) after 20 h and 60 h passed to show the impact of environmentally assisted cracking while the substrate cracking in long term crack growth behavior. For switching to air from dry nitrogen, the lid was removed from the stage and the nitrogen gas pumping was stopped at the same time therefore the sample was immediately exposed to air. The crack growth rates were measured after the required crack spacing was confirmed to involve or eliminate the interaction from neighboring cracks while optical imaging tracked through the observation area (region with a radius of 13.6 mm). To isolate the crack growth not to interact with neighboring cracks, 0.58% strain was required not to increase the density of cracks. The external-load-assisted channel crack growth technique was applied to achieve this condition without difficulty^23^. The density of cracks was also calculated by counting the number of propagated cracks in the observation area. Additionally, the crack configuration was specified from scanning electron microscope (SEM) images (Hitachi SU8230) and some of the specimens were further investigated by creating samples for imaging using a focused ion beam (FIB) (Nova FIB Micromanipulator, 30 kV 1–3 nA for FIB and 5 kV 0.4 nA for SEM) on where the channel crack is located. Au/Pd was sputtered (10 nm) on tested specimen before FIB to reduce charging effects. A 30 by 30 μm^2^ area was etched up to 10 μm deep.

### Mechanical characterization of SiN_x_ film, poly (ethylene terephthalate) substrate and polyimide substrate

Several mechanical properties are required to quantify the driving force for channel crack propagation. As mentioned previously, nanoindentation (Hysitron triboindenter) was used to measure the modulus of the SiN_x_ film *E*_*f*_. A 1 μm thick PECVD SiN_x_ on a Si substrate was indented to a depth of 100–300 nm. The microtensile stage was used to obtain uniaxial tensile properties of the PET and PI substrates. Table 1 summarizes all the related mechanical properties mentioned above.

**Table 1:** Mechanical properties of PECVD SiN_x_ film and PET/PI substrate.

Mechanical properties (units)	PECVD SiN_x_ film	Mechanical properties (units)	PET substrate	Mechanical properties (units)	PI substrate
*E*_*f*_ (GPa)	123 ± 5.8	*E*_s_^*^ (GPa)	4.47 ± 0.25	*E*_s_^*^ (GPa)	8.35 ± 0.19
*v*_f_	0.253	σ_0_ (MPa)	90.6		
*E*_f_^*^ (GPa)	131 ± 6.2	*D* (hr^−1^)	80		
ε_*res*_ (%)	−0.15 ± 0.02	*n′*	20		
		σ_y_ (MPa)	50 × (1 + 1.74ε_p_)		

### Numerical model

PET was modeled as an elastic-viscoplastic material, while SiN_x_ a purely elastic material. For PET, the elastic strain rate ${\dot{\varepsilon }}_{{\rm{e}}}$ is linearly proportional to the stress rate $\dot{\sigma }$ as

$${\dot{\varepsilon }}_{{\rm{e}}}=\dot{\sigma }/{E}_{s}$$

where *E*_*s*_ is the elastic modulus; a Cowper-Symonds overstress power law was applied to calculate the plastic strain rate.

4 $${\dot{\varepsilon }}_{p}=D{(\frac{\sigma }{{\sigma }_{Y}({\varepsilon }_{p})}-1)}^{n^{\prime} }$$

where *D* and *n*′ are the material constants, *σ*_Y_(*ε*_p_) is the yield stress that depends on the total yield strain *ε*_p_. These material properties are summarized in Table 1. Since no debonding was observed during experiments under subcritical loading, perfect bonding was assumed between the SiN_x_ thin film and PET substrate. Due to the large elastic mismatch between SiN_x_ thin film and PET substrate, large stress gradients may develop in PET parts underneath the cracks. To capture such large stress gradients in the PET substrate, a dense mesh scheme was employed for PET underneath the cracks. The growing crack in the PET substrate was modeled as an opening wedge. Penetration depth of the crack in the PET substrate can markedly affect the driving force of crack extension in the SiN_x_ thin film, due to a loss of substrate constraints. In our previous work^22^, we showed that the driving force change originating from the viscous response of polymer substrate is negligible. Specifically, PET relaxation at 0.95% applied strain resulted in less than 1% of *G* value change over 30 min and this amount of change will decrease and become negligible as the relaxation goes to a steady-state value over time. Therefore, the elastic-viscoplastic substrate behaves like an elastic substrate and each *G* value at different depths of substrate cracking does not involve the viscous substrate effect at the subcritical value of applied strain. All the numerical simulations were performed using ABAQUS 6.13^35^.

## References

[1] Zhang S, Xue W, Yu Z (2015). Moisture barrier evaluation of SiOx/SiNx stacks on polyimide substrates using electrical calcium test. Thin Solid Films.

[2] van Assche FJH (2014). On the intrinsic moisture permeation rate of remote microwave plasma-deposited silicon nitride layers. Thin Solid Films.

[3] Majee S, Geffroy B, Bonnassieux Y, Bourée J-E (2014). Interface effects on the moisture barrier properties of SiNx/PMMA/SiNx hybrid structure. Surface and Coatings Technology.

[4] Rochat G, Fayet P (2012). Characterization of Mechanical Properties of Ultra-thin Oxide Coatings on Polymers by Uniaxial Fragmentation Tests. Journal of Adhesion Science and Technology.

[5] Andringa AM (2015). Low-Temperature Plasma-Assisted Atomic Layer Deposition of Silicon Nitride Moisture Permeation Barrier Layers. ACS applied materials & interfaces.

[6] Bulusu A (2015). Engineering the mechanical properties of ultrabarrier films grown by atomic layer deposition for the encapsulation of printed electronics. Journal of Applied Physics.

[7] Li, F. M. *et al*. 18.3: Flexible Barrier Technology for Enabling Rollable AMOLED Displays and Upscaling Flexible OLED Lighting. *SID Int. Symp. Dig. Tech. Pap*. **44** (2013).

[8] Ok, K.-H. *et al*. Ultra-thin and smooth transparent electrode for flexible and leakage-free organic light-emitting diodes. **5**, 9464, 10.1038/srep09464 (2015). 10.1038/srep09464 PMC4379502 25824143

[9] Bae MS, Park C, Shin D, Lee SM, Yun I (2017). Effects of mechanical stresses on the reliability of low-temperature polycrystalline silicon thin film transistors for foldable displays. Solid-State Electronics.

[10] Choi, G.-M. *et al*. Flexible Hard Coating: Glass-Like Wear Resistant, Yet Plastic-Like Compliant, Transparent Protective Coating for Foldable Displays. *Adv Mater* **29**, 10.1002/adma.201700205 (2017). 10.1002/adma.201700205 28295731

[11] Kim I (2017). Selective Light-Induced Patterning of Carbon Nanotube/Silver Nanoparticle Composite to Produce Extremely Flexible Conductive Electrodes. ACS Applied Materials and Interfaces.

[12] Lee, M.-T. *et al*. Achieving a foldable and durable OLED display with BT.2020 color space using innovative color filter structure. 10.1002/jsid.533 (2017).

[13] Lee SM, Shin D, Yun I (2017). Degradation mechanisms of amorphous ingazno thin-film transistors used in foldable displays by dynamic mechanical stress. IEEE Transactions on Electron Devices.

[14] Yoon J (2017). Superflexible, high-efficiency perovskite solar cells utilizing graphene electrodes: Towards future foldable power sources. Energy and Environmental Science.

[15] Baumert EK, Pierron ON (2012). Fatigue properties of atomic-layer-deposited alumina ultra-barriers and their implications for the reliability of flexible organic electronics. Appl. Phys. Let..

[16] Sadeghi-Tohidi F, Samet D, Graham S, Pierron O (2014). Comparison of the cohesive and delamination fatigue properties of atomic-layer-deposited alumina and titania ultrathin protective coatings deposited at 200Â Â°C. Sci. Technol. Adv. Mater..

[17] Jen S-H, Bertrand JA, George SM (2011). Critical tensile and compressive strains for cracking of Al2O3 films grown by atomic layer deposition. Journal of Applied Physics.

[18] Jen S-H, Lee BH, George SM, McLean RS, Carcia PF (2012). Critical tensile strain and water vapor transmission rate for nanolaminate films grown using Al2O3 atomic layer deposition and alucone molecular layer deposition. Applied Physics Letters.

[19] Miller DC (2009). The mechanical robustness of atomic-layer- and molecular-layer-deposited coatings on polymer substrates. Journal of Applied Physics.

[20] Andersons J (2008). Evaluation of toughness by finite fracture mechanics from crack onset strain of brittle coatings on polymers. Theoretical and Applied Fracture Mechanics.

[21] Leterrier Y (2003). Durability of Nanosized Oxygen-barrier Coatings on Polymers. Prog. Mater. Sci..

[22] Kim, K. *et al*. Environmentally Assisted Cracking in Silicon Nitride Barrier Films on Poly(ethylene terephthalate) Substrates. *ACS applied materials & interfaces*, 10.1021/acsami.6b06417 (2016). 10.1021/acsami.6b06417 27643813

[23] Kim K, Graham S, Pierron ON (2017). Note: A single specimen channel crack growth technique applied to brittle thin films on polymer substrates. Rev Sci Instrum.

[24] Hutchinson JW, Suo Z (1992). Mixed mode cracking in layered materials. Advances in applied mechanics.

[25] Beuth J (1992). Cracking of thin bonded films in residual tension. International Journal of Solids and Structures.

[26] Dundurs J (1969). Discussion:“Edge-bonded dissimilar orthogonal elastic wedges under normal and shear loading”(Bogy, DB, 1968, ASME J. Appl. Mech., 35, pp. 460–466). Journal of applied mechanics.

[27] Thouless MD, Li Z, Douville NJ, Takayama S (2011). Periodic cracking of films supported on compliant substrates. Journal of the mechanics and physics of solids.

[28] Douville NJ, Li Z, Takayama S, Thouless MD (2011). Fracture of metal coated elastomers. Soft Matter.

[29] Thouless M (1990). Crack spacing in brittle films on elastic substrates. Journal of the American Ceramic Society.

[30] Delannay F, Warren P (1991). On crack interaction and crack density in strain-induced cracking of brittle films on ductile substrates. Acta metallurgica et materialia.

[31] Kang D (2014). Ultrasensitive mechanical crack-based sensor inspired by the spider sensory system. Nature.

[32] Wright, D. C. *Environmental stress cracking of plastics*. (iSmithers Rapra Publishing, 1996).

[33] Xia ZC, Hutchinson JW (2000). Crack patterns in thin films. Journal of the mechanics and physics of solids.

[34] Huang R, Prévost JH, Huang ZY, Suo Z (2003). Channel-cracking of thin films with the extended finite element method. Engineering Fracture Mechanics.

[35] ABAQUS/Standard 6.13, User’s Manual, SIMULIA, Providence, RI. (2010).

